# Prolonged survival by combination treatment with a standardized herbal extract from Japanese Kampo-medicine (Juzentaihoto) and gemcitabine in an orthotopic transplantation pancreatic cancer model

**DOI:** 10.3389/fonc.2024.1454291

**Published:** 2024-12-11

**Authors:** Joanna Napp, Paulina Siebel, Hans Rausch, Kenny Kuchta, Thomas Efferth, Frauke Alves, Volker Ellenrieder, Silke Cameron

**Affiliations:** ^1^ Clinic for Gastroenterology and Gastrointestinal Oncology, University Medical Center Göttingen (UMG), Göttingen, Germany; ^2^ Translational Molecular Imaging, Max Planck Institute for Multidisciplinary Sciences (MPI-NAT), Göttingen, Germany; ^3^ Institute of Diagnostic and Interventional Radiology, University Medical Center Göttingen (UMG), Göttingen, Germany; ^4^ Phytochem Reference Substances, Neu-Ulm, Germany; ^5^ Research Unit for Far Eastern Medicine, Department of Vegetation Analysis and Phytodiversity, Albrecht-von-Haller Institute for Plant Sciences, Georg-August-University Göttingen, Göttingen, Germany; ^6^ Institute of Pharmaceutical and Biomedical Sciences, Department of Pharmaceutical Biology, Johannes Gutenberg Universität Mainz, Mainz, Germany; ^7^ Clinic for Hematology and Medical Oncology, University Medical Center Göttingen (UMG), Göttingen, Germany

**Keywords:** Kampo medicine, Juzentaihoto, gemcitabine, pancreatic ductal adenocarcinoma (PDAC), orthotopic transplantation PDAC mouse model, murine KPC pancreatic tumor cells, macrophages, tumor microenvironment

## Abstract

Pancreatic ductal adenocarcinoma (PDAC) is characterized by its poor prognosis. Traditional Japanese herbal medicine (Kampo), such as Juzentaihoto (a standardized combination of 10 herbal extracts), has shown immune modulatory effects, modulation of microcirculation, and amelioration of fatigue. It is administered to patients to prevent deterioration of cachexia and counteract side effects of chemotherapy. The effect of Juzentaihoto with or without standard chemotherapy (Gemcitabine) on survival and tumor microenvironment was studied in an immunocompetent pancreatic cancer mouse model. Following tumor development ±12 days after orthotopic implantation of murine pancreatic cancer cells (KPC) into the pancreas of C57BL/6 mice, the mice were treated with Gemcitabine, Juzentaihoto, their combination (Gem/Juz) or NaCl (Ctr.). Combination treatment significantly prolonged survival (+38%) of tumor bearing mice, compared to controls as well as Gemcitabine or Juzentaihoto monotherapy. Macrophage (CD68+) infiltration in pancreatic tumors was significantly enhanced in Gem/Juz – treated animals, compared with controls (p < 0,001), with significant increases of both, macrophages (CD68+) and for lymphocytes (CD45+), especially at the tumor front. *In vitro*, Juz- or Gem/Juz-treated KPC tumor cells secreted significantly more macrophage-chemoattractant cytokines, e.g., CCL2, CCL20, and CXCL2, whilst Juz- and Gem/Juz-treated macrophages (MH-S) secreted cytokines of the M1 phenotype, e.g., IL6, TNF-α, and IL12. It has been shown that tumor cells recruit and polarize macrophages towards tumor-associated macrophages (TAM). Our results indicate a change in macrophage polarization which not only induced anti-tumor immune-cell activity and cytokine release, but also suggests amelioration of Gemcitabine efficacy as DNA-analogue and as partial antitumor antigen. We propose that the increased survival of tumor bearing mice after Gem/Juz combination treatment is due to the restored cytotoxicity of Gemcitabine and changes in the tumor-microenvironment - induced by Juzentaihoto - such as an increased number of M1 macrophages.

## 1 Introduction

Pancreatic adenocarcinoma (PDAC) is characterized by an aggressive phenotype and an extremely poor prognosis. Due to the frequently asymptomatic onset of the disease, patients are usually presenting with locally advanced - or metastatic disease with less than 20% eligibility for initial resection. Most patients experience local or systemic disease recurrence, resulting in an overall 5-year survival rate of less than 9% (1). Partial resistance to chemotherapeutic drugs and/or radiation therapy is common (2).

Chronic inflammatory processes and microcirculatory abnormalities accompany the development and growth of neoplastic tissue (3). This leads to a dense tumor stroma consisting of extracellular matrix (ECM) and cellular components such as cancer-associated fibroblasts (CAF), immune cells, especially macrophages, and endothelial cells. Tumor-associated macrophages (TAM) engage in bidirectional interactions with cancer cells. TAM are highly versatile and can be polarized into the M1-like pro-inflammatory phenotype that activates an immune response against the tumor and the M2-like immunosuppressive phenotype that promotes tumor immunity and progression (4, 5). M1/M2 phenotyping represents, however, only two extremes of the observed macrophage profiles (5).

Balancing inflammatory and redox-mechanisms, amelioration of microcirculation as well as wound-healing and nutrition have always been indications for herbal medicine (6). Once the body constitution deteriorates and cachexia sets in, a point of no return has been reached, forcing dose reduction or even termination of therapy (7). Learning how to manage these accompanying phenomena could significantly improve patients’ outcomes.

In Japan, traditional herbal medicine, Kampo, has been integrated into the modern medical system. Traditional prescriptions are standardized and controlled using GMP- and GCP-guidelines. Pharmaceutical and medicinal research is performed on an academic level and by pharmaceutical companies, besides trained clinical use. Together with the long-term experience over centuries, Kampo medicines – such as Juzentaihoto - can be used safely alongside Western medicine.

Juzentaihoto is covered by the Japanese National Health insurance and administered to support anti-tumor therapy, to alleviate side effects and in palliative cancer care to improve body constitution and appetite. This is particularly beneficial for patients with gastrointestinal cancers (8–10) and in particular for pancreatic cancer (6, 11–16). In China, this ancient Kampo-preparation is called *Shi-Quan-Da-Bu-Tang* and in Korea, *Sipjeondaebotang*.

Anti-cachexia are based - amongst others - on the activity of combined herbal extracts such as ginseng (*Panax ginseng*) or glycyrrhizae (*Glycyrrhiza uralensis*) radix on the mTOR-pathway, the STAT-pathway and melatonin (6) as well as ghrelin-enhancing properties (7).

As pancreatic cancer is the most aggressive gastrointestinal tumor, standardized Kampo extracts, especially Juzentaihoto with its immunomodulatory effects are of interest alongside chemotherapy (6, 7, 11, 17). It has been shown that Juzentaihoto increased leucocyte cell counts after chemotherapy (11) whilst protecting against myelosuppression (18). It also activated peritoneal macrophages against tumor cells and prevented liver- or lung metastasis (19, 20) in cell culture. The immune-enhancing effect of Juzentaihoto is involved in the prevention of metastasis, as this effect was abolished in mice with T-cell deficiencies (20).

Here, we show that the combination of Gemcitabine-based chemotherapy with the traditional herbal prescription Juzentaihoto prolonged the survival of pancreatic tumor-bearing mice. As the decrease of tumor size by Gemcitabine monotherapy did not translate into prolonged survival, we propose that Juzentaihoto-induced activation of tumor-associated macrophages not only induced anti-tumor immune-cell activity and cytokine release but also ameliorated Gemcitabine efficacy.

## 2 Materials and methods

### 2.1 Juzentaihoto extracts

Juzentaihoto consists of: *Panax ginseng* radix 3 g, *Atractylodes lancea* rhizome 4 g, *Poria cocos* sclerotium 4 g, *Glycyrrhiza uralensis* radix 2 g, *Angelica acutiloba* radix 4 g, *Paeonia lactiflora* radix 3 g, *Cnidium officinale* rhizome 3 g, *Rehmannia glutinosa* radix 4 g, *Astragalus membranaceus* radix 3 g, and *Cinnamomum cassia* cortex 3 g (**Supplementary Figure S1A**) (21). The single herbs have been imported from Toshimoto, Japan and provided by the “Kronen-Pharmacy,Wuppertal”.

For animal experiments, aqueous extracts of Juzentaihoto were freshly prepared from the above-named mixture of raw drugs: They were boiled for 30 min in water to a final volume of 400 ml shortly before use. For mice treatment, the final extracts were cooled down, filtered using common paper tea filter bags and changed 3 times per week to drink ad libitum. Whilst the leading components of Juzentaihoto are well characterized by HPLC (22), its efficacy relies on the combination of its multiple active ingredients such as components from Paeonia Radix (Paeoniflorin, and Albiflorin), Glycyrrhizae Radix (Isoliquritin, Liquiritin, Isoliquiritigenin, Glycyrrhizin and Formononetin), Cinnamomi Cortex (Cinnamaldehyde), and i.e. Angelicae Radix (Ligustilide and Xanthotoxin). Immunpharmacologically active polysaccharides have also been described, including pectins and pectic polysaccharides (23). In our experiments, we analyzed the combined effects, following the traditional application of Juzentaihoto.

For cell-culture experiments, the aqueous extract was lyophilized and re-diluted in cell-culture medium with 1% DMSO.

The quality of the extract was analyzed by HPLC (**Supplementary Figures S1B, C**). In brief: The aqueous extract of Juzentaihoto was filtered and used in comparison to the reference substance of the corresponding herbal drugs. Of each solution 125 µl were injected. For visual control, UV-spectra were employed. The HPLC from Merck-Hitachi (7000 D) was used with a corresponding HPLC-column [Merck (250 x 4 mm) type Lichrospher (100 RP18 5 µm)] and a precolumn (RP18 4 × 4 mm). The detection was performed by a diode array (210 nm) with a flow rate of 1 ml/min and a temperature of 20°C. Recording was done for about 100 min. The mobile phase contained acetonitril (0.05%), phorsphoric acid (1.5 ml + 2.5 ml aqua dest.) and methanol. The size of the membrane filter was 0.45 µm (Ø 3 mm, Schleicher & Schüll) with a Chromafil^®^ GF/PER-45/25 Macherey nail (1.0/0.45 µm Ø 25mm), and with a syringe filter of 0.45 µm (Ø 4 mm) for single use.

### 2.2 Cell lines and cell culture conditions

The murine pancreatic cancer KPC cell line (KPCbl6, clone 2.2) derived from the LSL-KrasG12D/+; LSL-Trp53R172H/+; Pdx-1-Cre (KPC) mouse model (24) was provided by Prof. V. Ellenrieder (University Medicine Göttingen). KPC cells were cultivated in high glucose Dulbecco’s modified Eagles medium (DMEM, Thermo Fisher Scientific, MA, Waltham, USA), supplemented with 10% fetal bovine serum (FBS Gold, PAA Laboratories Gold) and 1% non-essential amino acids (NEAA-100 X, Thermo Fisher Scientific, MA, Waltham, USA).

The murine alveolar macrophage MH-S cell line (CRL-2019, ATCC) was cultivated in complete Roswell Park Memorial Institute medium (RPMI, Thermo Fisher Scientific, MA, Waltham, USA) 1640, supplemented with 10% fetal calf serum (FCS) (Gibco) and 0.05 mM 2-mercaptoethanol (25).

All cells were maintained in culture at 37°C in a humidified atmosphere of 5% CO2.

### 2.3 Treatment efficacy *in vitro*

KPC cells were plated in a 96-well plate at a concentration of 5,000 cells per well and allowed to attach for ~24 h.

Afterwards, the medium was replaced with 100 µl of fresh medium supplemented with increasing concentrations of either Gemcitabine (HEXAL; 0, 1, 5, 10, 20, 40, 60, 80, 100, 125, 150, and 200 nM), or Juzentaihoto (water extract diluted 1:10, 1:20, 1:50, 1:75, 1:100, 1:200, 1:300, 1:400, 1:500, and 1:1000 in cell culture medium supplemented with 1% dimethyl sulfoxide (DMSO) or with a combination of both. Control cells received corresponding volumes of water and 1% DMSO. The experiment was performed in duplicate.

Plates were placed in the Incucyte Live-Cell Imaging and Analysis Instrument (Sartorius). Phase contrast images (two images per well) were collected every hour with a 10× objective. The IC50 values were calculated after 72 h of incubation - a time when untreated cells reached 100% confluence.

### 2.4 RNA extraction and real-time PCR

KPC and MH-S cells were plated in a 6-well plate at a concentration of 1 × 10^6^ cells per well (or 0.5 × 10^6^ cells per well, respectively, and allowed to attach for ~24 h. Afterwards, medium was replaced with 2 ml of fresh medium supplemented with either Gemcitabine (HEXAL; 80 nM), or Juzentaihoto (water extract diluted 1:20 in cell culture medium supplemented with 1% DMSO or a combination of both. Water (1:20) and DMSO (1%) were used as controls.

After 24 h, the cells were washed with phosphate buffered saline (PBS), and total RNA was extracted with the TRIzol^®^ reagent (Thermo Fisher Scientific, MA, Waltham, USA). After addition of 200 µl chloroform, the tubes were vortexed and incubated at room temperature for 5 min. After a centrifugation step at 13,500 rpm at 4°C for 15 min, the supernatant was separated and 500 µl isopropanol was added. After 10 min incubation at room temperature probes were centrifuged (13,500 rpm at 4°C) for 30 min. The pellet was then diluted in 750 µl 75% ethanol and vortexed. After 5 min centrifugation (13,500 rpm at 4°C), the supernatant was discharged and a second washing step with 75% EtOH was performed, followed by centrifugation. The pellet was then dried and 30 µl of RNA-free water was added on ice.

For cDNA synthesis the iScript cDNA Synthesis Kit was used according to the manufacturer’s instructions (Bio-Rad Laboratories, Feldkirchen, Germany), with the following program: 25°C (5 min), 46°C (20 min), 95°C (1 min), then cooling to 4°C. After adding 80 µl, RNA-free water cDNA was stored at -20°C.

Quantification of the mRNA was performed by relative quantification using Platinum™ SYBR™ Green qPCR SuperMix-UDG (Invitrogen, Darmstadt, Germany) in the following concentrations: 5 µl SYBR Green, 3.5 µl H2O dest., 0.25 µl forward primer, and 0.25 µL reverse primer. Then, 1 µl cDNA was added and RT-PCR (96 well-plate) was performed in triplicates. **Supplementary Table S1** shows the list of the primers used (Invitrogen, Darmstadt, Germany).

PCR conditions were set as follows: 50°C for 2 min, 95°C for 2 min, and 45 cycles of 95°C/15 sec and 60°C/30 sec. As housekeeping gene, Ribosomal Protein, Large, P0 (RPLP0) was used, which was checked for stability. For data analysis, Step one PluS software (v2.3) was employed.

### 2.5 Cytokine/chemokine array

For treatment experiments, KPC and MH-S cells were plated at a concentration of 1 × 10^6^ or 0.5 × 10^6^ cells per well, respectively, in a 6-well plate and allowed to attach for ~24 h. Afterwards, the medium was replaced with 2 ml of fresh medium supplemented with either Gemcitabine (HEXAL; 80 nM), Juzentaihoto (water extract diluted 1:20 in cell culture medium supplemented with 1% DMSO) or a combination of both. Medium with addition of water (1:20) and DMSO (1%) was used as control. After 24 h treatment, supernatants were collected, centrifuged for 5 min at 14.000 rpm, stored at -20°C and used for cytokine profiling.

Cytokine profiling was performed using the Mouse XL Cytokine Array Kit (Proteome ProfilerTM Array; R&D systems) with 1 ml of cell culture supernatants (KPC and MH-S cells), according to the manufacturers protocol. Data were quantified using ImageJ software (FIJI) (26) and are shown as an average intensity normalized to the reference spots after background subtraction.

### 2.6 Vimentin-cadherin immunofluorescence staining

Aliquots of 50,000 cells/well were grown on poly-L-lysine coated coverslips in a 24-well plate for 24 h, followed by a 24 h treatment with Gemcitabine, or Juzentaihoto, or a combination of both, or water/DMSO as a control as described above in the Cytokine/chemokine array section. Afterwards, the cells were washed briefly with PBS, fixed with ice-cold 4% formaldehyde for 15 min, washed again with PBS (3 × 10 min), and permeabilized in 1% Triton X-100 in PBS for 10 min followed by three PBS washes. Subsequently, the cells were blocked in 0.5% bovine serum albumin (BSA) in PBS for 1 h at room temperature (RT) and incubated with the mouse monoclonal anti-vimentin antibody (Santa Cruz Biotechnology, E-5, 1:200) and the rabbit monoclonal anti-E-cadherin antibody (Cell signaling, 24E10, 1:100) in 0.5% BSA for 1 h at RT. After three washes with 0.1% Tween20 in PBS for 10 min each, the cells were incubated for 1 h at RT with fluorescence-labeled secondary antibodies: anti-mouse (Thermo Fisher, A-11003, Alexa Fluor 546) and anti-rabbit (Thermo Fisher, A-21206, Alexa Fluor 488), each diluted 1:500 in 0.5% BSA in PBS. Nuclei were stained for 10 min with Hoechst 33342 diluted 1:1.000 in PBS. The cover slips were mounted with Aquatex aqueous mounting medium (Merck). Confocal fluorescence microscopy was performed using the Leica SP2 system. The following excitation and emission settings were used: for Alexa Fluor 488: λEx = 488 nm and λEm = 500-550 nm, for Alexa Fluor 546: λEx = 561 nm und λEm = 570-650 nm and for DAPI: λEx = 405 nm and λEm= 410-480 nm. Images were analyzed using ImageJ software (FIJI) (26).

### 2.7 Animal studies

All animal experiments were performed in accordance with German Animal Welfare Act regulations and were approved by the Local Ethics Office of Lower Saxony (LAVES; license no. 33.9-42502-04-18/2953; Oldenburg, 22.11.2018). Experiments were performed on 10-16 weeks old C57BL/6 male mice (Charles River Laboratories), housed in ventilated cages, and allowed food and drink (water or Juzentaihoto) ad libitum.

**Figure 1:**
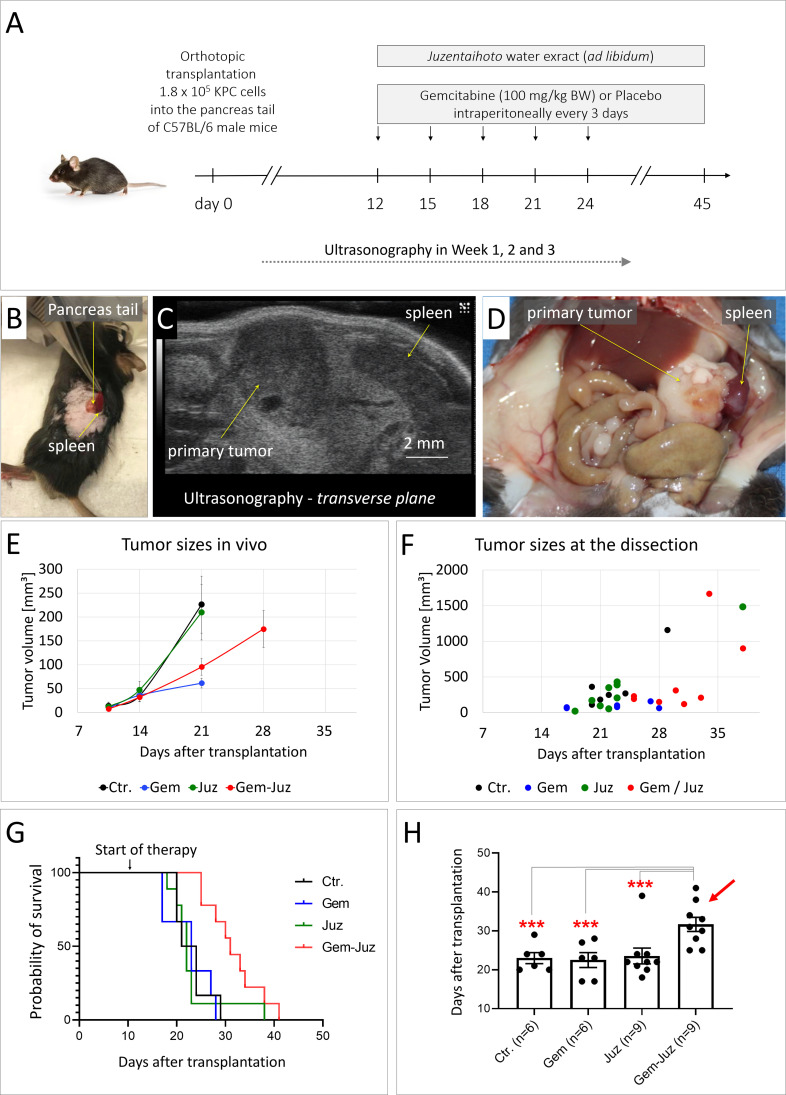
Effect of Gemcitabine and Juzentaihoto on tumor growth and overall survival. **(A)** Treatment scheme (controls received placebo = 0.9% NaCl) with **(B)** orthotopic syngenic transplantation of murine pancreatic cancer KPC cells into the pancreatic tale of C57BL/6 mice. **(C)** Example of ultrasound visualization of the primary tumor; here, the transversal plane is shown. **(D)** At autopsy: visualization of the primary tumor and spleen with metastasis. **(E)** Tumor growth monitored with ultrasonography over time. Error bars correspond to the SEM. Note, that the average tumor size at week 4 could only be calculated for the group treated with Gem-Juz, as the survival time was shorter in the other groups. **(F)** Tumor sizes at dissection. **(G)** Kaplan-Meyer analysis of survival within the different groups. **(H)** Average survival within the treatment groups, displayed as histogram, ***p ≤ 0.05.

30-45 min prior to the surgery, mice received analgesic buprenorphine (intraperitoneally, 0.1 mg/kg body weight (BW); 10 µl/g BW). After induction of anesthesia with isoflurane (4%, maintenance 2.5%), mice received the analgesic Rimadyl^®^ (subcutaneously; Carprofen 5 mg/kg BW, 5 µl/g BW, Zentiva) and the abdominal skin and peritoneum were opened with a ~5 mm incision in the left upper abdomen. The pancreas was exposed and ~1.8 × 10^5^ cells resuspended in 40 µl of culture medium/matrigel (1:1) were slowly transplanted into the pancreatic tail with an insulin syringe. The pancreas was reintroduced into the abdominal cavity and peritoneum and skin were sutured. For analgesia, mice received Rimadyl for 2 days after surgery. Mice were weighted and inspected three times per week for general condition, and abdominal palpation was performed to detect tumor formation. Tumor size was measured weekly with small animal high-resolution ultrasound as previously described (27). Briefly, isoflurane (~2%) anesthetized mice were placed on a heated stage and the abdominal area was depilated. Ultrasonography (US) was performed using the Visual Sonics Vevo 2100 High Resolution Ultrasound System equipped with the Vevo 2100 MicroScan Transducer MS-550-D (22-55 MHz). Sagittal and transversal planes were imaged (**Figures 1A–C**).

After development of visible (US) pancreatic tumor (day 12), mice were randomized into four groups: placebo (n = 6), Gemcitabine (Gem) (n = 6), Juzentaihoto (Juz) (n = 9), and Gemcitabine plus Juzentaihoto (Gem/Juz) (n = 9). Fresh Juzentaihoto decoction was prepared 3 times per week as described above and administered ad libitum in place of drinking water. Gemcitabine (HEXAL) was diluted in 0.9% NaCl (100 mg/kg BW; 5µl/g BW) and administered intraperitoneally (i.p.) three times per week. Placebo-treated mice received equal volumes of saline injections (NaCl 0.9%). Treatment was continued until each individual animal reached the defined endpoint. Endpoint criteria were defined as 20% BW loss, general morbidity, lethargy, lack of social interaction or development of ascites. Kaplan-Meier survival analysis was performed using GraphPad Prism (9.5.0).

During autopsy (**Figure 1D**), each tumor was measured with a caliper and tumor volume was calculated based on a simplified formula: length × width × high × 0.5. Tumor and remaining pancreas were collected, fixed in 4% paraformaldehyde (PFA, Sigma) for 24 h, dehydrated in ascending concentrations of ethanol and embedded in paraffin. Tissue sections 2.5 μm were processed for histochemistry, i.e., Hematoxylin-Eosin (H&E), Masson-Trichrom (MTS) and Berlin Blue staining, immunohistochemistry and immunofluorescence.

### 2.8 Histochemistry and immunohistochemistry

Note that all staining was performed on sequential tissue sections. For the representative histological images, tumors were selected from mice sacrificed at similar times for all treatment groups (approximately 3 weeks) after transplantation. This means that even for the groups with prolonged average survival, a mouse was selected that died approximately 3 weeks after transplantation. The selection of areas in the consecutive microscopic images aimed to include the tumor tissue, the invasion front, and if possible any remaining pancreatic tissue.

For all staining, 2.5 μm thick tissue sections (tumor with adjacent pancreas) were deparaffinized, rehydrated and pretreated at 98°C for 20 min in citrate buffer (pH 6.0, Dako). Histochemical staining was performed on formalin-fixed and paraffin-embedded tissue sections as previously described (28).

For immunhistochemistry, endogenous peroxidase activity was inhibited on tissue sections with 3% H2O2 and unspecific binding sites were blocked with SEA BLOCK blocking buffer for 20 min. Slices were stained with primary antibodies against the following antigens: CD68 (Abcam, ab125212, 1:500), MHC II (1:100, Biolegend), CD163 (Abcam, ab182422, 1:500), Lipocalin (Abcam, ab 216462, 1:2,000), CD45 (Abcam, 208022, 1:1,000), CD3 (Abcam, ab5690, 1:100), CD4 (Abcam, ab237722, 1:1,000), CD8 (1:500, Abcam, ab228965), Ki-67 (Cell Marque, 275R-14, 1:200), and α-SMA (Dako, Clone 1A4, 1:250, RRID: AB_2335694), overnight at 4°C.

Finally, all slides were incubated with a corresponding HRP-labeled secondary antibody (Histofine; ready-to-use), stained with AEC (3-amino-9-ethylcarbazole) substrate (Pharmingen), washed with dH2O, counterstained with H&E, mounted with aqueous mounting medium (Aquatex), and dried overnight at RT. The images were acquired using the Axiovert 200M microscope (Leica) equipped with an AxioCamHR camera and processed with ImageJ (version 2.9.0/1.53t) (26).

### 2.9 Cell counting of immune cells in histological slides

In order to quantify macrophage infiltration, the stained slices were initially scored in a blinded fashion by two independent observers (score 1-5, with 1 = none to minor macrophage infiltration and 5 = high infiltration). For further quantification and validation of macrophage (CD68-positive) and leucocyte (CD45-positive) numbers, automated cell counting was performed using ImageJ (26). Briefly, the following areas were separately analyzed for each mouse: i) tumor center, ii) tumor border (invasion front), and iii) remaining pancreatic tissue. Four images were randomly recorded for each of these areas, resulting in a total of 12 analyzed areas per mouse. The analyzed regions measured 708 × 530 µm. Images were captured using an Axiovert 200M microscope with a 20× objective. For each staining, a macro was developed and visually inspected to ensure the accurate selection of various cell types, focusing on the identification of positively stained cells. Color deconvolution was used to generate a black-and-white picture, a threshold was defined, and noise was reduced. A watershed system allowed to distinguish neighboring cells, and cells were counted automatically using Image J. The median number of positive cells per area (µm²) per mouse was calculated automatically with CSV-tables.

### 2.10 Molecular docking analysis

The 3D-structure of the interleukin 6 receptor (PDB ID: 7dc8) was retrieved from the RCSB Protein Data Bank (rcsb.org). Hetero-atoms and water molecules were deleted, polar hydrogen atoms were added, missing atoms were repaired, Kollman charges were added and finally saved in PDBQT format on AutoDockTools 1.5.6 (https://ccsb.scripps.edu/mgltools/). The prescreening was performed with PyRx AutoDock VINA (blind docking mode) to monitor docking to the entire surface of IL6R, and the Lamarckian algorithm of AutoDock VINA was chosen for the defined docking mode). The Lamarckian algorithm was used to analyze the docking poses and binding energies as described (29, 30). Three independent docking calculations were conducted with 25,000,000 energy evaluations and 250 runs by using the Lamarckian genetic algorithm. Visual Molecular Dynamics software was used for visualization (https://www.ks.uiuc.edu/Research/vmd/).

### 2.11 Statistical analysis

The statistical analysis including Kaplan-Meier survival analysis and graphic display were done with GraphPad Prism (9.5.0). The values for the cell counts are expressed as median ± SE (standard error).

Nested one-way ANOVA was conducted for the analysis, comparing the four groups with three areas for each group (tumor, tumor border, and pancreatic tissue). If only one specific tissue type was analyzed, an ordinary 1-way ANOVA was performed. For Image J, 5% of the outliers were excluded.

For experiments with three biological and three technical replicates (Western Blot and qRT-PCR), the mean value for the biological replicate includes the mean of the technical replicates.

P values ≤ 0.05 were considered statistically significant. P-values ≤ 0.05, ≤ 0.01, ≤ 0.001, and ≤ 0.0001 are denoted with *, **, ***, and ****, respectively. Non-significant results are indicated as ‘ns’.

## 3 Results

The composition of the Juzentaihoto formulation was confirmed by HPLC. The chromatograms showed the specific peaks of Juzentaihoto with visualization of the significant lead compounds via the retention time and the UV7VIS spectra (**Supplementary Figure S1**) (22).

### 3.1 Survival is independent of tumor growth

To investigate the effect of Juzentaihoto *in vivo*, the aqueous extract of Juzentaihoto was administered *ad libitum* to mice with orthotopic PDAC tumors, either alone or in combination with Gemcitabine. Gemcitabine was applied intraperitoneally at a dose of 100 mg/kg BW every three days.

At the time of tumor cell transplantation, the average animal weight was 25.1 ± 1.5g. At day 8/9 after orthotopic transplantation of KPC cells, when the average tumor size measured by ultrasonography (US) was 10.4 +/- 18.4 mm^3^, mice were divided into 4 treatment groups; group 1: control (n = 6), group 2: Gemcitabine (n = 6), group 3: Juzentaihoto (n = 9) and group 4: Gemcitabine and Juzentaihoto (n = 9). The average tumor size in the control group was 13.7 ± 20.9 mm³, in the Gemcitabine group 9.4 ± 13 mm³, in the *Juzentaihoto* group 11.8 ± 27.8 mm³ and in the Gemcitabine and *Juzentaihoto* group 7.7 ± 7.9 mm³. The average animal weight was: 25.4 ± 0.8 g in the control, 25.6 ± 1.8 g in Gemcitabine, 24.9 ± 1.6 g in *Juzentaihoto* and 25.2 ± 1.8 g Gemcitabine and *Juzentaihoto* groups. All treatments started at day 12 (**Figure 1A**). All treatments started at day 12 (**Figure 1A**).

In the Juzentaihoto-treated groups, the water was replaced by the Juzentaihoto decoction, which was well-tolerated and drunk ad libitum. On average, a mouse drinks around 15 ml of fluid per 100 grams of its body weight daily, which corresponds to approximately 3.75 ml of fluid for a 25 g mouse. This amounts to about 150 g/kg body weight per mouse. There were no symptoms of dehydration, diarrhea or weight loss that could be attributed to Juzentaihoto treatment. Nevertheless, and as expected during disease progression, mouse weight decreased individually with time-to-death. However, no significant difference in body weights was observed within the different groups with high inter-mouse variability (**Supplementary Figure S2**).

Juzentaihoto alone had no effect on tumor growth, with average tumor sizes almost identical within the control- and the Juzentaihoto-groups. As shown in the **Figure 1E**, at week 3 (~ at the time of the 4^th^ Gemcitabine dose), the average tumor size measured with US in control- and Juzentaihoto- treatment groups were 225.9 ± 103.5 and 209.7 ± 115.5 mm³, respectively. Gemcitabine chemotherapy on the other hand, led to a significantly decelerated tumor growth, whether used alone (61.6 ± 20.1 mm³) or in combination with Juzentaihoto (95.5 ± 52.2 mm³). This is even more prominent at the later stages of the treatment, as shown in the **Figure 1F**, where tumor sizes at the time of dissection for each individual animal are presented.

The decrease in primary tumor growth however did not translate into survival (**Figures 1G, H**). At the time of autopsy, metastases had remained local (*i.e*., infiltration into the spleen). Distant metastases into the liver were not observed. While Gemcitabine monotherapy effectively reduced tumor growth, it failed to prolong mice survival. Mean overall survival in the control group was 23.0 days (± 3.5) and in the groups treated with Gemcitabine or Juzentaihoto monotherapy 22.5 days (± 4.7) and 23.6 days (± 6.1), respectively. Only supportive treatment with Juzentaihoto extract together with Gemcitabine increased life expectancy significantly: Mice in the combination treatment arm showed an average survival of 31.7 days (± 5.5), which was ~ 38% longer than the average survival of controls.

### 3.2 Histochemical analysis of tumor response

**Figure 2:**
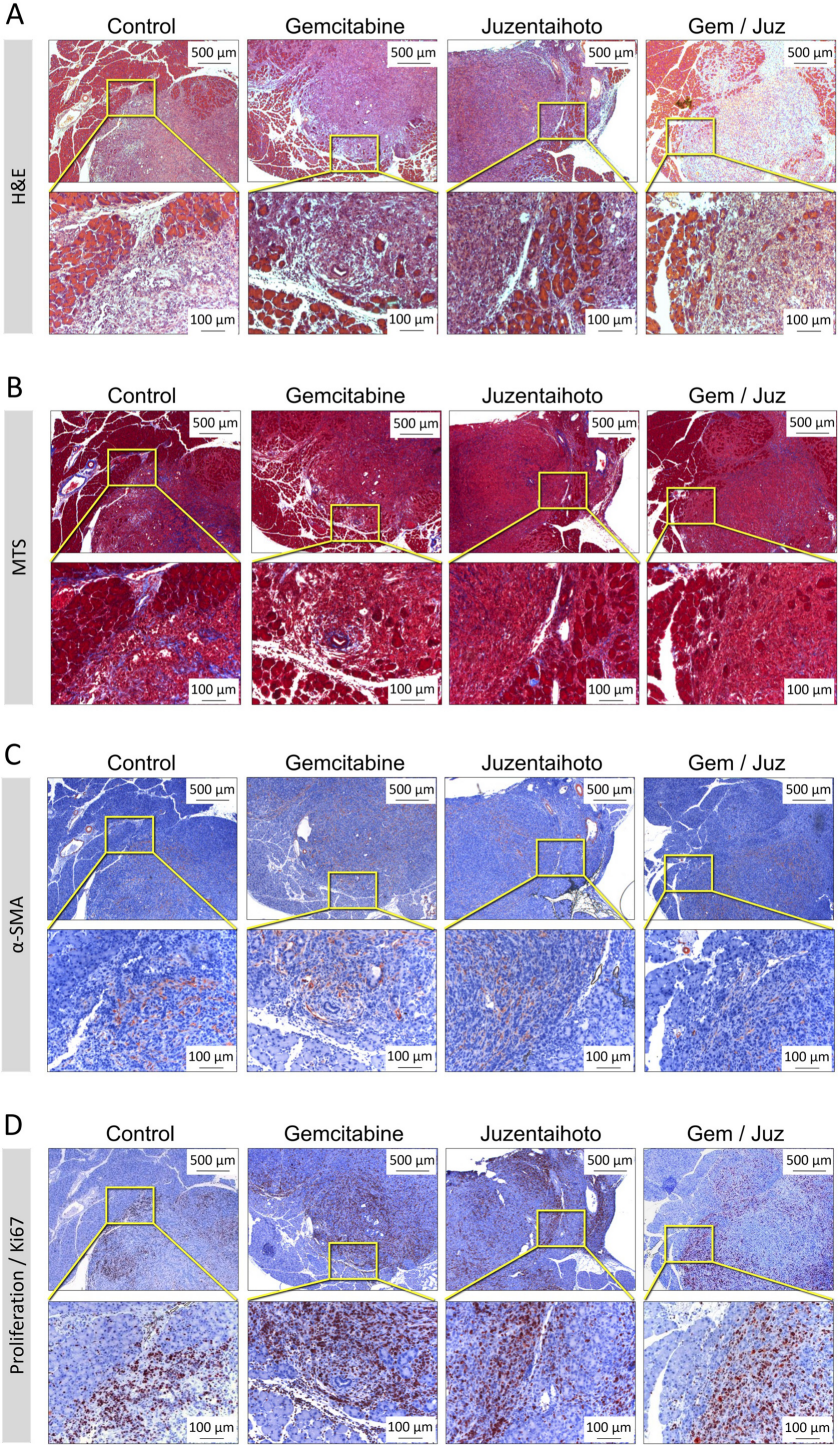
Representative microscopic images of the general tumor morphology among treatment groups performed on serial sections. **(A)** H&E staining, **(B)** Masson Trichrome staining (MTS), **(C)** IHC of α-smooth muscle actin (α-SMA) and **(D)** Ki-67 staining. Images show tumor slices taken from representative mice approximately 3 weeks after transplantation. Scale bars correspond to 500 µm (upper row) and 100 µm (lower row), respectively.

In H&E staining all treatment groups showed infiltration by tumor cells into the pancreatic tissue with separation of single acini from the lobe structure and an inflammatory infiltrate (**Figure 2A** shows representative images, from about 3 weeks after transplantation). With combination-treatment (Gem/Juz) the tumor itself appeared looser with surrounding acini more contiguous than in Gemcitabine treatment alone.

While pancreatic cancer in patients is usually characterized by a dense fibroblastic tumor stroma, the mesenchymal tumor stroma in our orthotopic KPC transplantation model appears to be negligible and not significantly differently between the four groups: the Masson Trichrom staining (MTS) revealed only a minor collagen deposition (**Figure 2B**). Also, only a moderate expression of alpha-smooth muscle actin (α-SMA) was observed within the tumor tissue, probably in myofibroblasts and small tumor vessels. (**Figure 2C**).

All tumors were highly proliferative, as confirmed by Ki-67 staining (which associates cell-cycle- dependent with various types of chromatin) (**Figure 2D**). Ki-67^+^ cells were preferentially located at the tumor periphery in all four groups. However, Ki-67^+^ cells appeared to be more prominent in tumors of mice treated with Gemcitabine as compared to the combination treatment (Gem/Juz).

### 3.3 Immunohistochemical analysis of the immune-cell microenvironment

**Figure 3:**
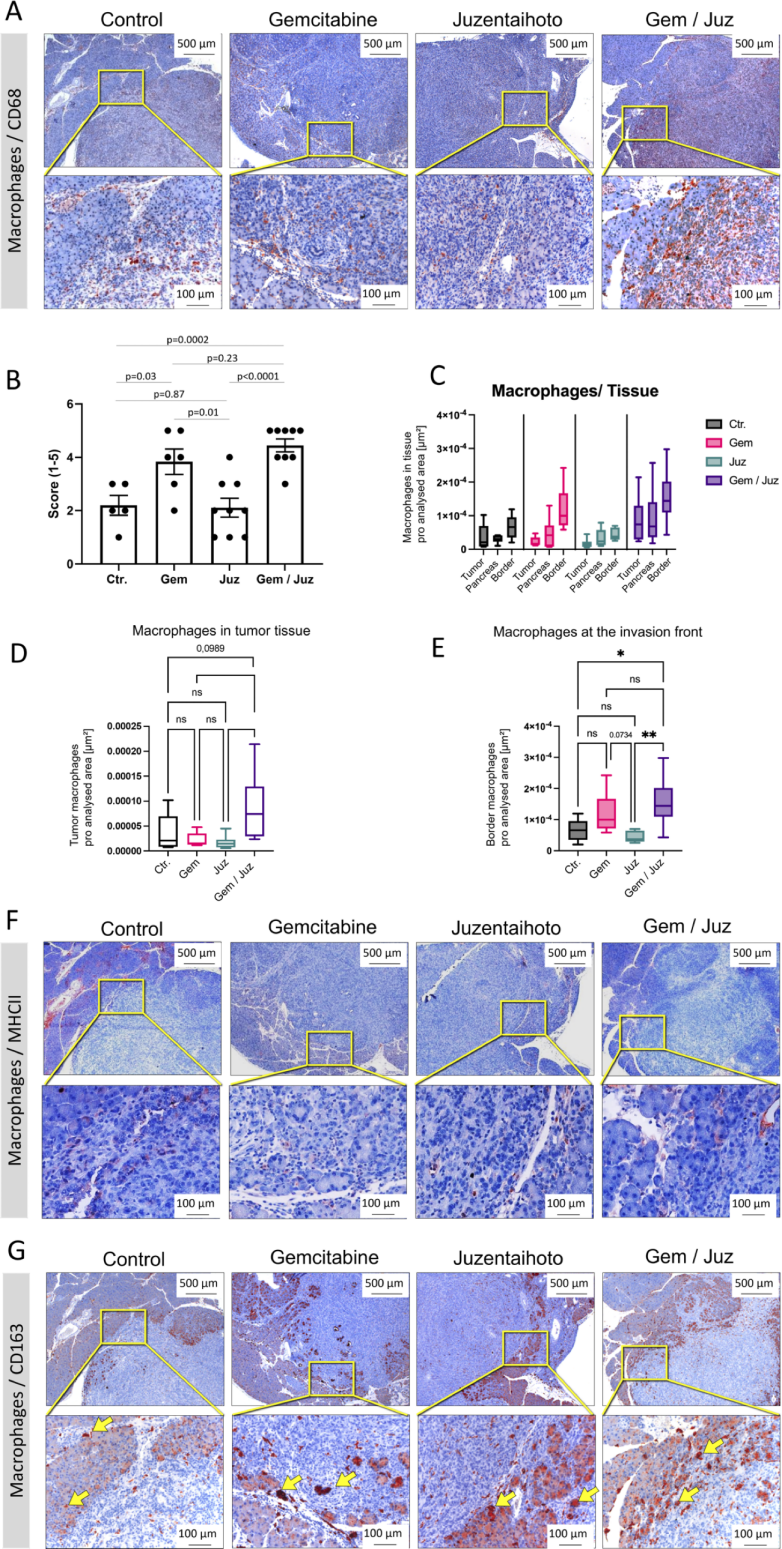
Macrophage infiltration of KPC tumors upon treatment of mice with Gemcitabine, Juzentaihoto, and their combination. Staining was performed on sequential slices. **(A)** CD68 staining of representative sequential tumor sections of the four treatment groups. Note the significant increase in CD68^+^ cells after combination treatment (Gem/Juz). **(B)** Scoring of CD68^+^ macrophages within the four groups (n = 6 for placebo and Gemcitabine and n = 9 for Juzentaihoto and Gem/Juz groups). **(C-E)** Image J-based quantifications and comparisons of the macrophage infiltration within the primary tumor, at the tumor border (infiltration front) and within the adjacent pancreatic tissue. Results confirm the significant increase of CD68^+^ macrophages after combination treatment (Gem/Juz), especially at the tumor border. **(F)** MHCII staining at the tumor infiltration front. Only scarce MHCII^+^ cells are visible, even at higher magnification (40x). **(G)** CD163 staining at the tumor infiltration front. Rather than depicting macrophages, the antibody stains the hemoglobin-haptoglobin complex of tumor-adjacent acinar cells (yellow arrows, see below). Scale bars correspond to 500 µm (upper row) and 100 µm (lower row), respectively. P-values ≤ 0.05 and ≤ 0.01 are denoted by * and **, respectively. Non-significant results are indicated as ‘ns’. P-values close to a significance level are displayed as numbers.

Immune microenvironment and immune cell infiltration of tumors is often closely related to clinical outcomes. To determine whether immune-cell infiltration in our KPC mouse model was influenced by the various treatments, sequential slides were stained with a macrophage detecting anti-CD68 antibody. Whilst Juzentaihoto alone did not significantly alter CD68^+^ macrophage infiltration, we observed an increase upon Gemcitabine treatment, especially at the tumor border, which was even higher in the Gem/Juz combination treatment (**Figure 3A**). In order to quantify macrophage infiltration within the tumor tissue, the stained slices were scored in a blinded fashion by two independent observers (score 1-5, with 1 = none to minor infiltration and 5 = high infiltration). As shown in **Figure 3B**, the scoring of CD68+ macrophages confirmed our qualitative observations and showed significantly higher scoring in the combination treatment when compared to control (p = 0.0002) and Juzentaihoto (p < 0.0001) groups. Increased macrophage infiltration was also observed after Gemcitabine monotherapy (in comparison to controls (p = 0.03)), with a clear, although not significant (p = 0.23) further increase after Gem/Juz treatment. CD68^+^ cells were further quantified using Image J (**Figures 3C–E**). Here, three areas were selected for quantification: tumor tissue, adjacent pancreatic tissue and the tumor margin at the transition to pancreatic tissue (invasion front). As depicted in **Figures 3C–E**, the digitized analysis corroborated our initial visual assessment, revealing a notable increase in CD68^+^ macrophages following combination treatment, particularly at the tumor border. Additionally, there was a discernible, although - at the border not statistically significant - increase observed with Gemcitabine monotherapy when compared to the control group.

As CD68 serves as a general macrophage marker, we proceeded to differentiate M2 macrophages. Thus, we performed IHC for MHC Class II and CD163. Visual evaluation of MHCII staining showed negligible numbers of MHCII^+^ cells without apparent differences within the four groups (**Figure 3F**). Similarly, CD163^+^ M2 macrophages were also found to be infrequent (**Figure 3G**). These findings suggest that the increased number of macrophages, especially at the tumor border, observed upon Gemcitabine and combination treatment predominantly consisted of M1 macrophages.

Besides being a marker for M2-macrophages, CD163 is known as high affinity scavenger receptor for the hemoglobin-haptoglobin complex (31). Scavenging of iron within the complex protects against its proinflammatory and potentially toxic effects. It is, thus, interesting to note that in our study, CD163 apparently stains heme complexes within tumor-adjacent acinar cells, rather than depicting individual immune cells (**Figure 3G**; arrows). This was confirmed when we stained for lipocalin-2, an iron sequestering protein (**Supplementary Figure S3A**). Gemcitabine especially seemed to trigger iron uptake, whilst free iron deposits (Berlin blue reaction) were scarce (**Supplementary Figure S3B**).

Staining for T-cells (CD3, CD45) as well as their subgroups: cytotoxic T-cells (CD8) and T-helper cells (CD4) did not show significant overall-changes throughout the tissue. The numbers of CD8^+^ and CD4^+^ cells were too minor for a meaningful sub-analysis. **Supplementary Figure S4** exemplarily shows CD45 immunostaining. For CD45^+^ lymphocytes at the tumor border, Image J-supported subgroup-analysis revealed a significant increase after Gemcitabine treatment (compared to controls).

Whilst Gemcitabine treatment inhibits tumor growth and affects lymphocyte numbers at the tumor border, its combination with Juzentaihoto seems to affect macrophage numbers, especially at the tumor front.

### 3.4 Behavior of KPC cells *in vitro* in response to Juzentaihoto and combination treatment

To assess cellular viability *in vitro*, KPC cells were treated for 72 h with increasing concentrations of Gemcitabine (0-200 nM), Juzentaihoto (1:10 – 1:1000), a combination of the two, or solvent alone as control. Cell growth was monitored over time using the Incucyte live cell imaging system, and the IC_50_ was calculated based on cell confluency after 72 h treatment (**Supplementary Figures S5A, B**). As in the mouse experiments, Juzentaihoto alone did not inhibit tumor cell growth. Gemcitabine and the combination of Gem/Juz however showed a strong and significant growth inhibitory effect on KPC tumor cells attributable to the cytotoxic effect of Gemcitabine, with IC_50_ of 13.5 nM and 21.6 nM, respectively.

With the aim of analyzing cellular differentiation in view of epithelial-to-mesenchymal transition within the various treatment groups and control, double fluorescent staining was performed using an antibody against E-cadherin for epithelial cells and vimentin, respectively for stromal/mesenchymal cells. As shown in the **Supplementary Figure S5C**, the majority of control KPC cells exhibited an epithelial phenotype, as evidenced by positive staining for E-cadherin, with fewer vimentin-positive mesenchymal cells. A similar morphology/phenotype was observed in KPC cells after 24 h Juzentaihoto treatment (1:20). Despite a significantly reduced cell viability observed in both Gemcitabine-treated (80 nM) and double-treated cells due to treatment, the surviving cells maintained their differentiation (with E-cadherin positivity) at this time (**Supplementary Figure S5C**).

### 3.5 Chemokine expression by tumor cells *in vitro*

To better understand the mechanisms responsible for macrophage infiltration and immune modulatory mechanisms, we analyzed chemokine expression by tumor cells *in vitro*. Consequently, a chemokine-cytokine array was performed after 24 h incubation of KPC cells with control, Gemcitabine (80 nM), Juzentaihoto (1:20), or both.

**Figure 4:**
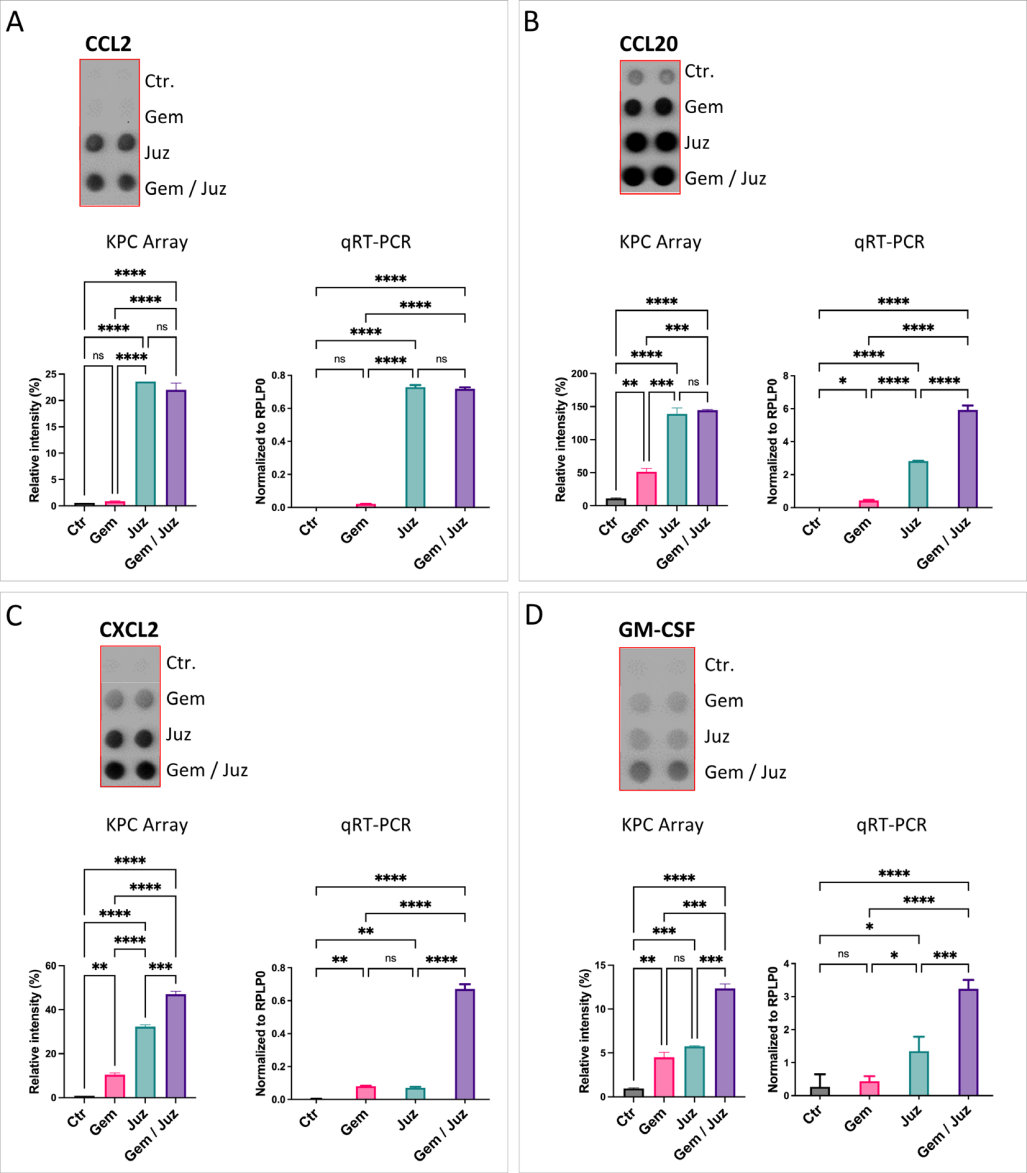
Expression and secretion of macrophage chemoattractant cytokines **(A)** CCL2, **(B)** CCL20, **(C)** CXCL2, and **(D)** GM-CSF by KPC cells upon treatment with Gemcitabine (80nM), Juzentaihoto (1:20 in medium with 1% DMSO), and their combination. Chemokine Arrays are shown as dot-blots (refer also to **Supplementary Figure S6**) and corresponding histograms after quantification. The expression of cytokines (CCL2, CCL20, CXCL2, and GM-CSF) was verified at the RNA level with qRT-PCR. Notably, Juzentaihoto treatment and combination therapy (but not Gemcitabine treatment alone) demonstrated a significant increase in the expression of macrophage chemoattractive/stimulating cytokines: **(A)** CCL2, **(B)** CCL20, **(C)** CXCL2, and **(D)** GM-CSF. P-values ≤ 0.05, ≤ 0.01, ≤ 0.001, and ≤ 0.0001 are denoted with *, **, ***, and ****, respectively. Non-significant results are indicated as ‘ns’.

Juzentaihoto treatment as well as the combination treatment (but not Gemcitabine treatment alone) showed a significant increase of the macrophage chemoattractant chemokines CCL2/MCP-1, CCL20/MIP3α, and CXCL2/MIP-2 in the supernatant of tumor cells (**Figures 4A–C**; **Supplementary Figure S6**). Furthermore, granulocyte macrophage colony stimulating factor (GM-CSF) was also increased by Juzentaihoto treatment and combination treatment, however to a lower extend (**Figure 4D**). These results suggest that following Juzentaihoto treatment, tumor cells may release chemoattractive cytokines, indicating a potential correlation with the observed increase in macrophage infiltration. The treatment with Juzentaihoto appeared to stimulate the secretion of signaling molecules by tumor cells, possibly contributing to the enhanced recruitment of macrophages to the tumor microenvironment.

To study, whether Juzentaihoto can also directly affect macrophages causing their activation, we performed cytokine expression analyses, incubating macrophages of the murine macrophage MH-S cell line with the different treatments (control, Gemcitabine, Juzentaihoto, or their combination).

### 3.6 Chemokine/cytokine expression by macrophages *in vitro*

**Figure 5:**
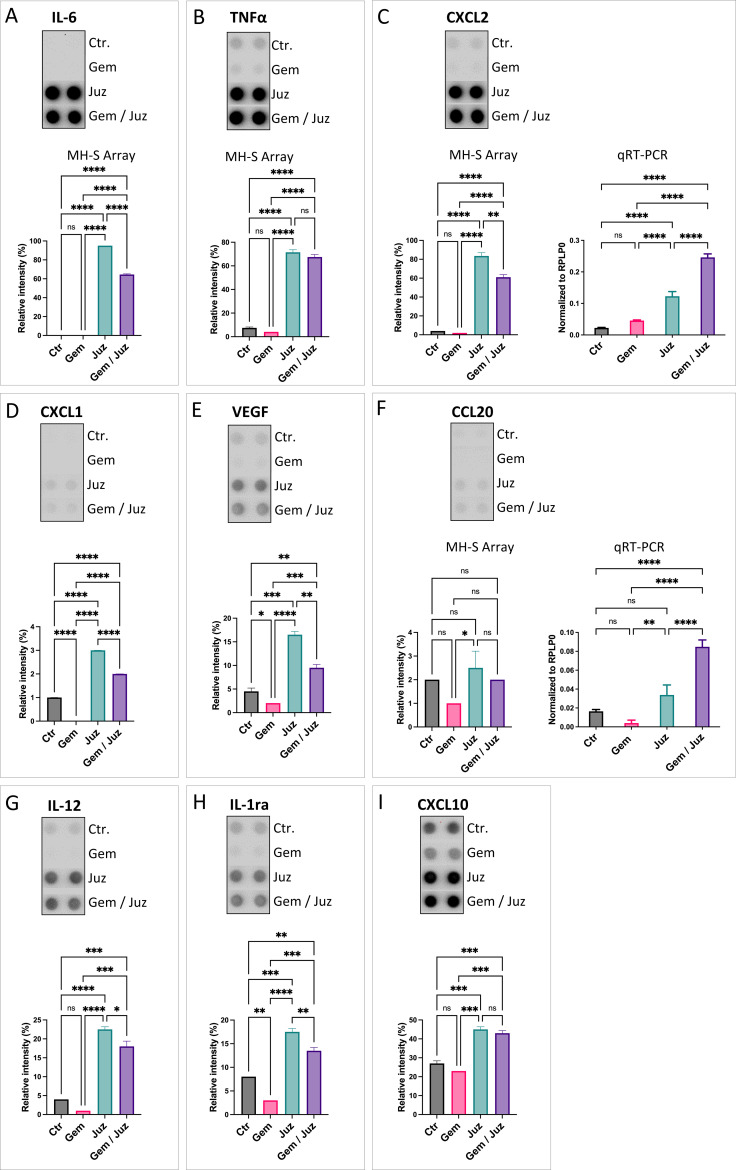
Chemokine secretion upon treatment of MH-S macrophages with Gemcitabine (80 nM), Juzentaihoto (1:20 in medium with 1% DMSO), and the combination of both. **(A)** IL-6, **(B)** TNF-α, **(C)** CXCL2/MIP2, **(D)** CXCL1/Gro1, **(E)** VEGF, **(F)** CCL20/MIP3, **(G)** IL-12, **(H)** IL-1ra, and **(I)** CXCL10. Chemokine arrays are shown as dot-blots and corresponding histograms after quantification. For C) and F) the protein expression was verified at the RNA level with qRT-PCR. P-values ≤ 0.05, ≤ 0.01, ≤ 0.001, and ≤ 0.0001 are denoted with *, **, ***, and ****, respectively. Non-significant results are indicated as ‘ns’.

As shown in **Figures 5A, B** (see also **Supplementary Figure S6**), the classical pro-inflammatory M1-cytokines IL-6 and TNF-α were increased upon macrophage-stimulation with Juzentaihoto or combination treatment (but not Gemcitabine alone). This strongly suggests, that Juzentaihoto treatment not only induces KPC tumor cells to produce macrophage attracting chemokines (**Figure 4**), but also acts on macrophages, reprogramming them to the M1 phenotype. Similar to the inflammatory stage of wound healing, we further showed an induction of CXCL2/Gro2/MIP2 and to a lesser extent CXCL1/Gro1 as well as CCL20/MIP3α by Juzentaihoto. More than CXCL1/Gro1, which also has pro-angiogenic properties, the pro-angiogenic factor VEGF itself was increased upon Juzentaihoto treatment (**Figures 5C–F**). Furthermore, IL-12 – a T-cell costimulatory factor (32) was induced – (**Figure 5G**). At the same time, cytokines which are involved in the prevention of overshooting immune reactions were also elevated by Juzentaihoto, such as Interleukin 1 response element (IL-1ra) which abrogates NF-κB activation, and the γ-interferon response element CXCL10/IP10 which is involved in anti-tumor activity (**Figures 5H, I**).

### 3.7 Molecular docking analysis

The 3D-structure of the interleukin 6 receptor (PDB ID: 7dc8) was retrieved from the RCSB Protein Data Bank (rcsb.org) to monitor docking to the entire surface of IL6R. Virtual comparison with an online databank showed that albiflorin, benzoylpaeoniflorin, and glycyrrhizin interact with the IL6 receptor.

## 4 Discussion

Tumor-stroma interaction influences tumor progression and its response to chemotherapy (33). The idea of a tumor as ‘wound that does not heal’ still holds.

Herbal medicine has always been employed to balance immune reactions as well as redox-mechanisms and ameliorate microcirculation and nutrition (6). The ancient prescription Juzentaihoto which has been used to treat cachexia for centuries, has - in recent years - played an increasing role in the supportive treatment of cancer patients, especially those with pancreatic cancer (6, 11–16). It is therefore intriguing to explore the effect of Juzentaihoto on survival and tumor microenvironment in the immune-competent KPC mouse transplantation model of pancreatic cancer, especially in the context of the chemotherapeutic agent Gemcitabine. KPC cells carry both, a mutation in the oncogene KRAS (LSL-KrasG12D/+) and in the tumor-suppressor gene P53 (LSL-Trp53R172H/+), which makes the tumor in our experimental model rather aggressive.

We showed that combination treatment of Gemcitabine and Juzentaihoto significantly prolonged survival of KPC tumor bearing mice not only, when compared to placebo-treated mice (+ 8.67 days; 37.7%), but also compared to mice treated with Juzentaihoto (+ 8.11 days) or Gemcitabine alone (+ 9.17 days). In cell culture, Gemcitabine (as chemotherapeutic agent) showed (alone or in combination with Juzentaihoto) the expected cell depletory effect on KPC tumor cells. Similarly, *in vivo* tumor growth was decreased in Gemcitabine-treated mice, as well as with combination treatment. The effect of Gemcitabine on tumor volume did not, however, translate into prolonged survival. We can conclude that the trigger of the combination treatment for a better survival rate must be located elsewhere, and it is self-suggestive to look into the tumor microenvironment.

Tumors shape their own microenvironment. The immune system responds to the tumor in the context of its microenvironment which varies according to tumor stage. To some extent, the genetic background of the tumor contributes (34). In this context, we formerly showed that expression of tumor-related oncogenes and loss of tumor-suppressor genes influence chemokine expression (35).

Pancreatic adenocarcinomas are generally considered non-immunogenic, displaying low infiltrates of cytotoxic T-lymphocytes (CTLs) along the invasive margin but not within the tumor core (36). This is in accordance with our observations, where T-cells (CD45^+^) were visible at the infiltration front and increased after Gemcitabine treatment – with the unmasking of tumor-epitopes. Recognition and killing of transformed cells by effector T-cells (NK and cytotoxic CD8^+^ cells) takes place in the early elimination phase, when the tumor is not-yet host to its own microenvironment (37). Accordingly, the numbers of CD8^+^ and CD4^+^ cells in our experiments were negligible.

Whilst the tumor modifies the microenvironment to limit the host response, the adaptive immune system reacts to tumor specific antigens. Tumor-associated macrophages (TAMs) can be of an activated M1 or an immune-suppressive M2 phenotype. Whilst the M1 phenotype secretes cytokines such as IL6, TNF-α, and CCL2 and is involved in the synthesis of reactive oxygen-species (ROS), the M2 phenotype seems to be better adapted at scavenging debris, promoting angiogenesis and tissue remodeling (38–40). This phenotype has thus been termed pro-tumorigenic. Halbrook, et al. showed that tumor-associated (M2) macrophages (TAM) release a spectrum of pyrimidine species. These include deoxycytidine. Gemcitabine differs from deoxycytidine only by two Fluorine atoms at the deoxyribose of the nucleoside cytidine (**Supplementary Figure S7**). Due to the similarity in molecular structure, Gemcitabine and deoxycytidine compete intracellularly for deoxycytidine kinase, the enzyme necessary for Gemcitabine activation. Halbrook, et al. showed that deoxycytidine blocks the cytotoxic effect of Gemcitabine dose-dependently. As a consequence, in an M2 microenvironment, Gemcitabine activation and activity as a chemotherapeutic drug decreases (41). Thus, tumor-associated (M2) macrophages not only scavenge debris and promote tumor growth, they also inhibit Gemcitabine activity (38–41). Whilst it is known that M2 macrophages are associated with poor prognosis (36, 42), they are also involved in tumor cell invasion (43). Macrophage activation to the anti-tumor M1 phenotype can explain the overall better prognosis in the combined treatment group with ameliorated efficacy of Gemcitabine.

It is known that tumor epitopes can be unmasked by chemotherapy which further leads to better macrophage recognition. Such antigens are derived from proteins involved in the DNA-damage response (44, 45). Accordingly, amino acid anchor-residue modifications and changes in peptide length render peptides to favor surface expression of alternative HLA-alleles with increased immunogenicity (46). As Gemcitabine unmasks epitopes important for macrophage recognition, it is intriguing to ask whether Gemcitabine would be more effective in an inflammatory background.

The presentation of exogenous antigens on MHC class I molecules is vital for the detection of cancer by immune cells (46). Our results are coherent with reports on histopathological findings from patients with pancreatic cancer, where a high density of macrophages at the tumor border was associated with an ameliorated response to chemotherapy (42). The authors propose that the number of macrophages should be taken into account when selecting patients for chemotherapy with Gemcitabine (42).

In our tumor mouse model, we observed a significant increase in CD68^+^ macrophages in response to combination treatment with Gemcitabine and Juzentaihoto. Accordingly, we showed in pancreatic cancer cells (KPC) *in vitro* an induction of monocyte/macrophage-chemoattractant cytokines such as CCL2/MCP1, CCL20/MIP3α, CXCL2/MIP2α and also GM-CSF by Juzentaihoto (and the combination). These results suggest that combination treatment induces macrophage chemotaxis and activation, ultimately leading to the induction of anti-tumor immunity (47).

Furthermore, Juzentaihoto (and its combination with Gemcitabine, but not Gemcitabine alone) increased the production and release of acute phase cytokines (IL-6, TNF-α) from macrophages, as well as of pro-inflammatory chemokines such as CXCL2/MIP2α/Gro2, and to a smaller extend CXCL1/Gro1 and CCL20/MIP3α. Juzentaihoto also increased interleukin receptor antagonist IL-1ra and CXCL10/IP10, thus suggesting the modulation of IL-1 and interferon-related immune reactions, preventing overshooting immune reactions.

**Figure 6:**
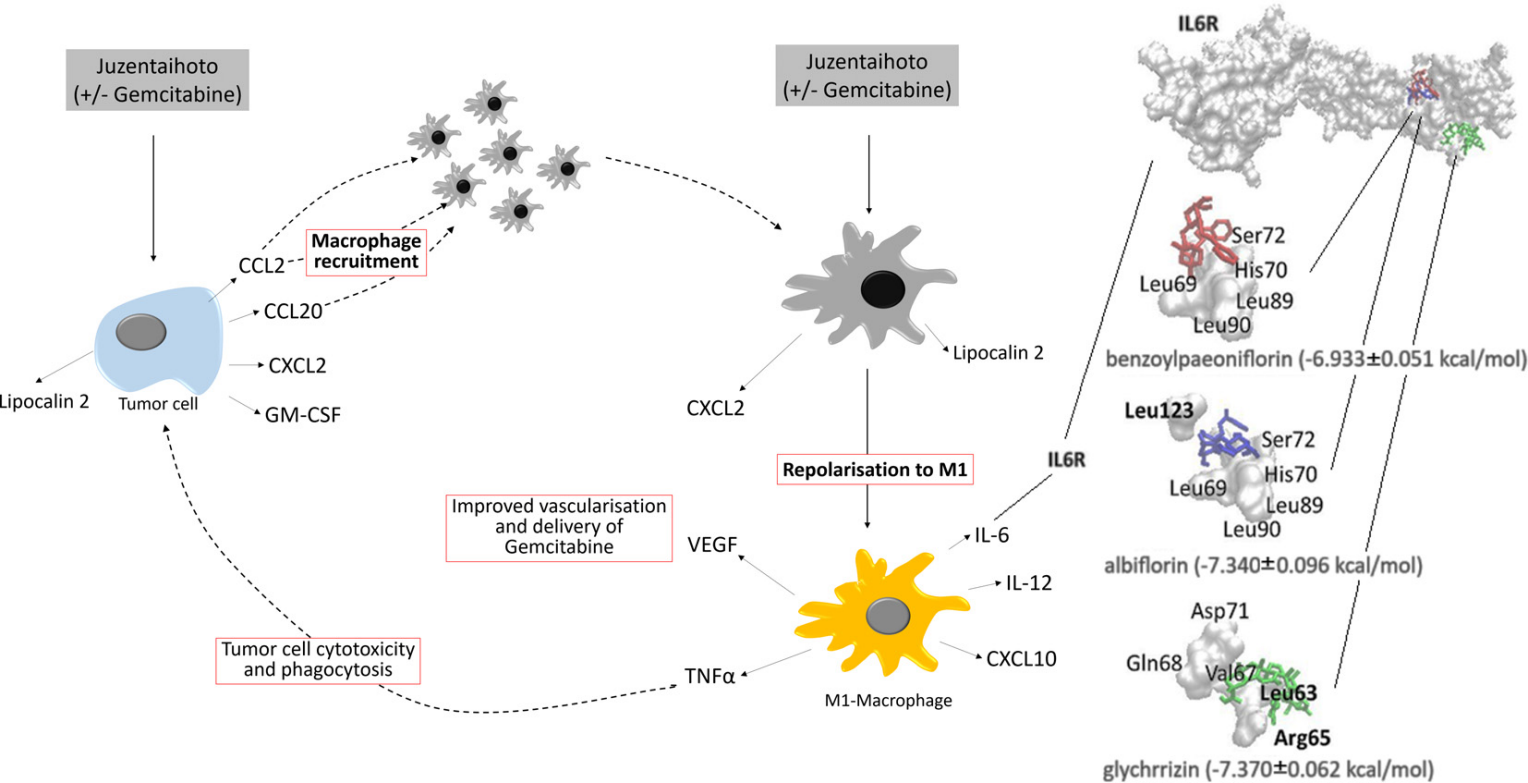
Postulated effect of Juzentaihoto in combination with Gemcitabine in a KPC model of pancreatic cancer. Left: Upon treatment with Juzentaihoto alone or in combination with Gemcitabine, but not Gemcitabine alone, pancreatic cancer cells release macrophage-chemoattractive cytokines (CCL2, CCL20, CXCL2) as well as macrophage colony stimulating factor (GM-CSF). Right: Upon treatment with Juzentaihoto (alone or in combination with Gemcitabine), macrophages are repolarized to the IL-6, IL-12, TNF-α and CXCL10-producing M1 macrophages known to have anti-tumoral properties. Note that IL-6 is increased upon Juzentaihoto-treatment. Using a virtual interaction map (far right) we showed that acting constituents of Juzentaihoto bound to the IL-6 receptor.

*In silico* research allows to predict interaction of the acting constituents of Juzentaihoto with various protein residues (**Figure 6**). Virtual comparison with an online databank showed that albiflorin, benzoylpaeoniflorin, and glycyrrhizin interact with the IL6 receptor. Like Gemcitabine, Juzentaihoto has various acting modalities. Whilst Juzentaihoto induces macrophages to produce cytokines like IL6 and Tumor necrosis factor (TNF- α) in our experiments, some of its active constituents (from ginseng and glycyrrhizae *radix*) hinder the inflammatory arm of IL6 signaling, thus preventing muscle wasting and cachexia (6). It has further been shown that Isoliquiritigenin, a flavonoid compound of Glycyrrhizae root blocks M2 macrophage-polarization (48). It is one column of the adaptogenic effect against cachexia (6), which shall be explored further.

Another double-edged sword is the amelioration of microcirculation. Whilst common strategies try to prevent neoangiogenesis in tumors by inhibition of VEGF, wound healing involves the amelioration of microcirculation. Also because of its fibrosis, pancreatic cancer is rather resistant to chemo- and radiotherapy. VEGF-inhibitors are thus not employed. However, it would be reasonable to ameliorate microcirculation in this context. We showed that Juzentaihot*o* enhances VEGF expression by macrophages. On the other hand, it has been shown that Juzentaihoto suppresses tumor-induced angiogenesis in B16 melanoma cells *in vivo* and *in vitro* (49). It follows that the significance of Juzentaihoto as pro- or antiangiogenic factor varies in view of the cellular background, *i.e*., immune- or tumor cell. Further research would be necessary in this regard.

From our study we conclude that Juzentaihoto-induced polarization of tumor-associated macrophages into the M1 phenotype not only induces anti-tumor immune-cell activity and cytokine release (such as TNF-α, IL6), it also ameliorates Gemcitabine efficacy in view of DNA-analogue as well as partial antitumor antigen.

Taken together, our results suggest that the combination treatment of Gemcitabine and Juzentaihoto changes the microenvironment of pancreatic cancer and thus prolongs survival. Our results support Japanese studies suggesting that Kampo medicine, especially Juzentaihoto, can be a supplementary treatment option, especially in pancreatic cancer.

## Data Availability

The original contributions presented in the study are included in the article/**Supplementary Material**. Further inquiries can be directed to the corresponding author.
