# Supplementary figures and images for: Prolonged survival by combination treatment with a standardized herbal extract from Japanese Kampo-medicine (Juzentaihoto) and gemcitabine in an orthotopic transplantation pancreatic cancer model
Source: Front Oncol. 2024 Dec 11;14:1454291. doi: 10.3389/fonc.2024.1454291 (PMC11669038; doi:10.3389/fonc.2024.1454291)

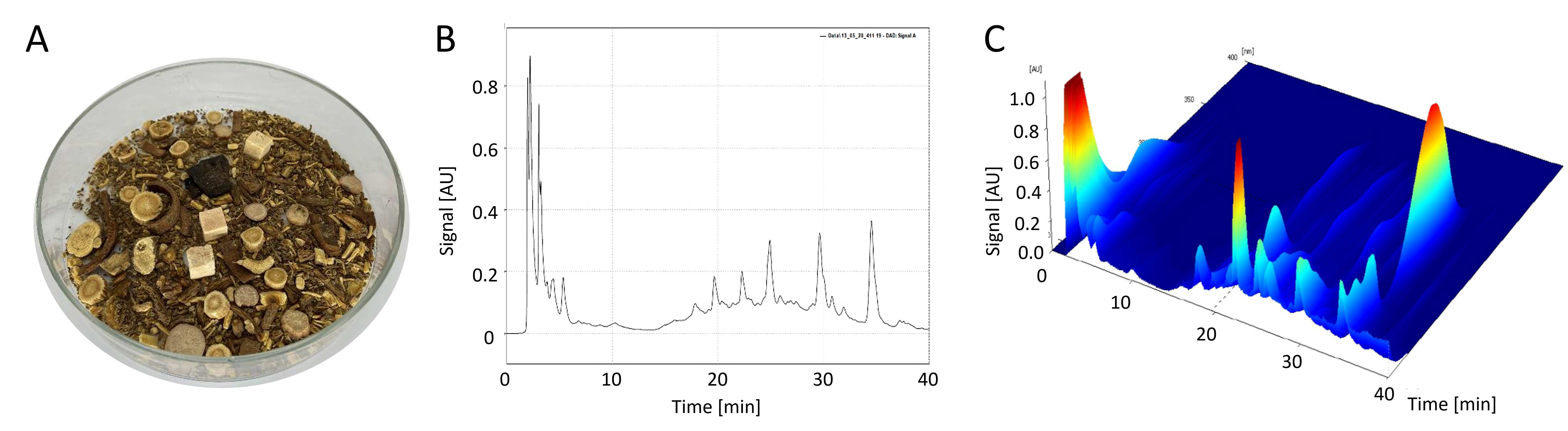

Supplement: Supplementary file 1 [file Image1.tif]

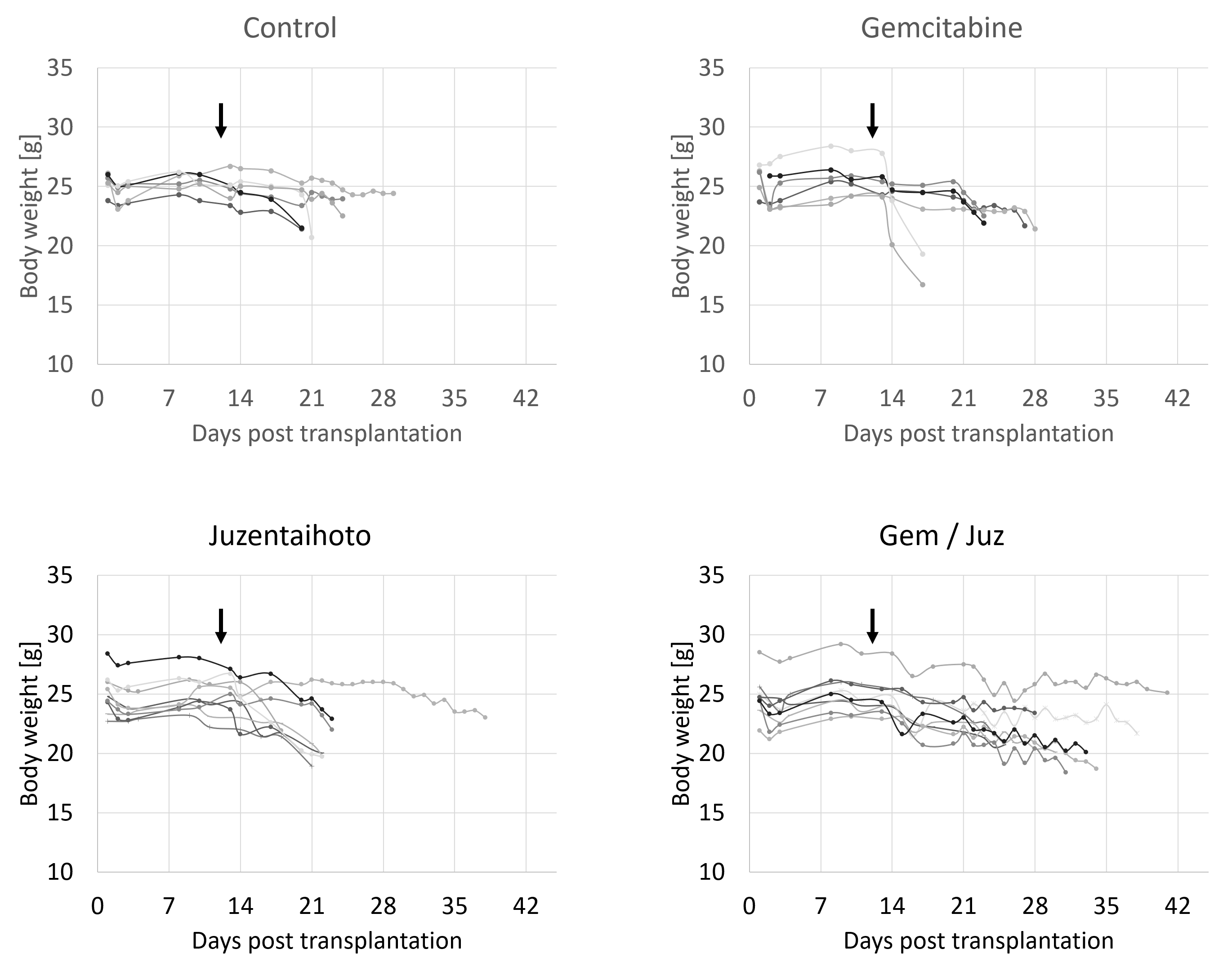

Supplement: Supplementary file 2 [file Image2.tif]

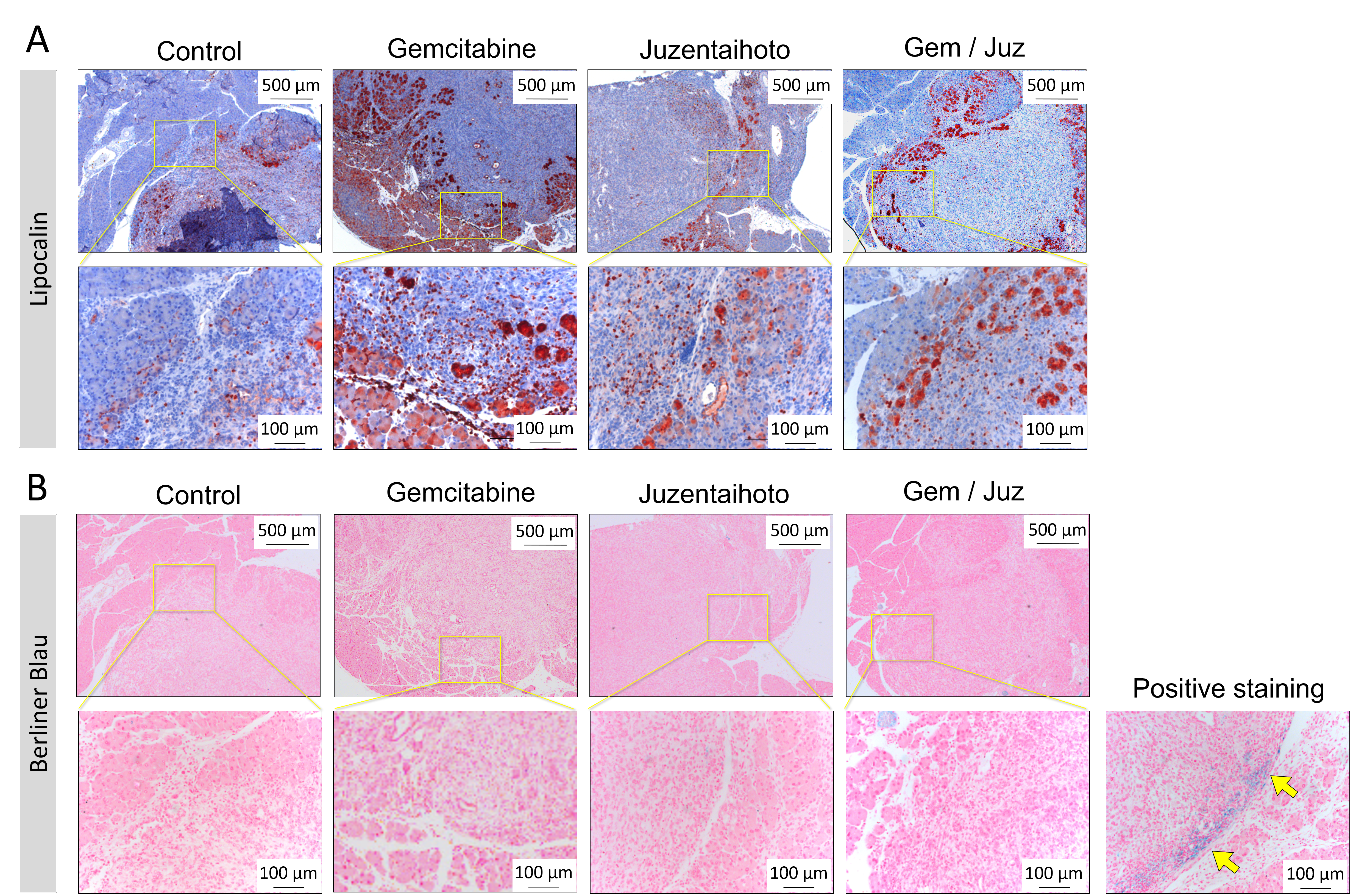

Supplement: Supplementary file 3 [file Image3.tif]

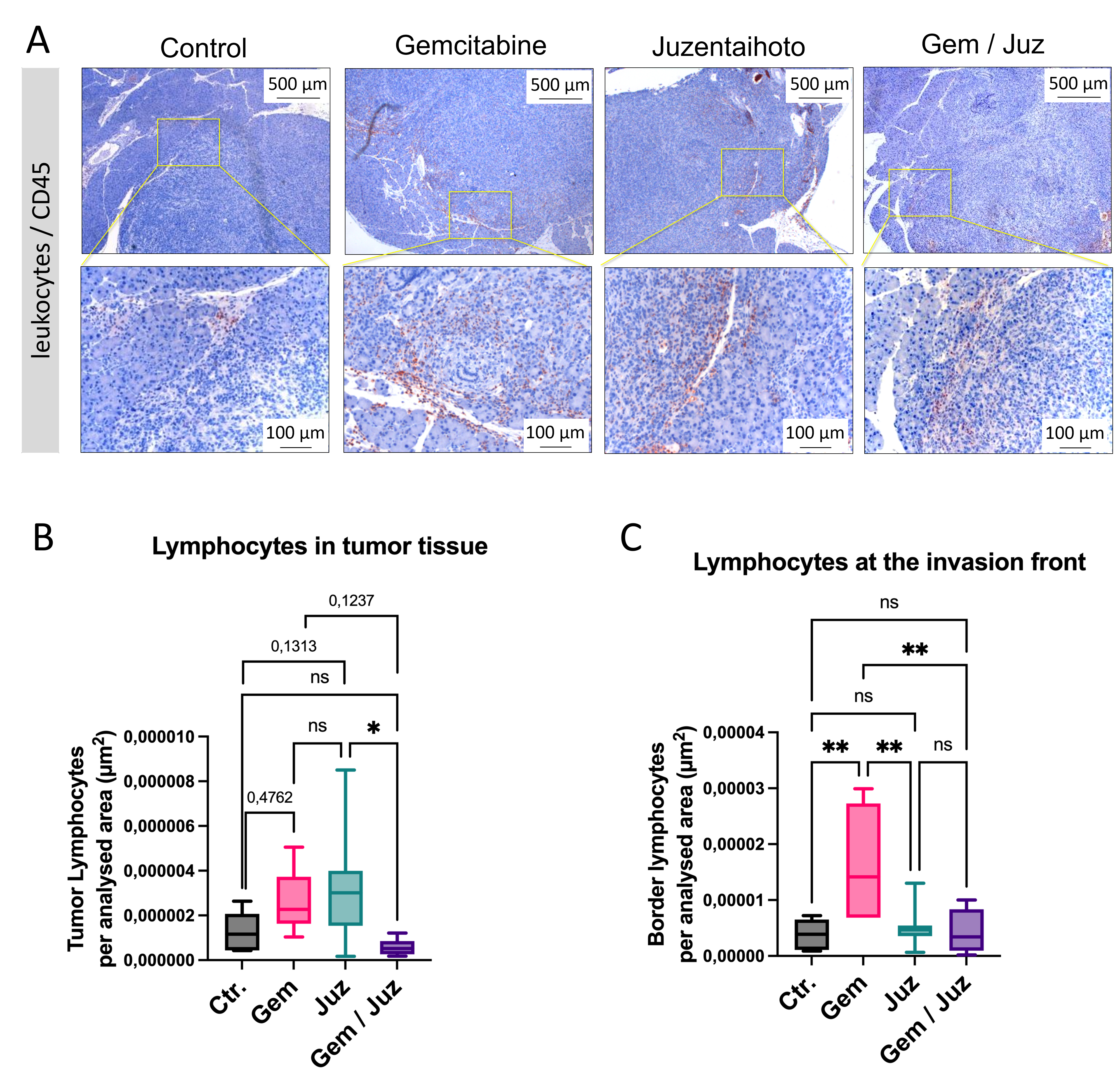

Supplement: Supplementary file 4 [file Image4.tif]

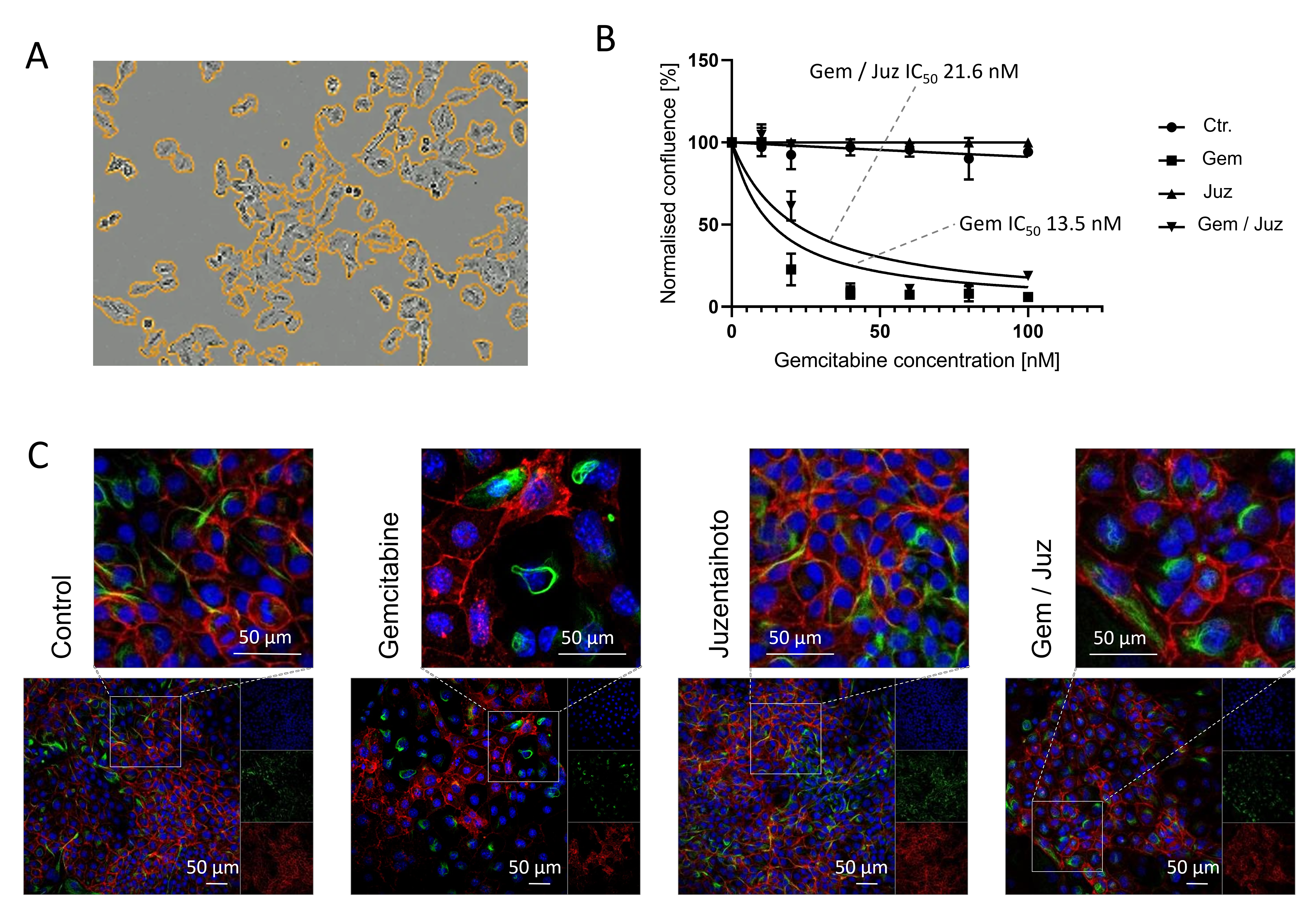

Supplement: Supplementary file 5 [file Image5.tif]

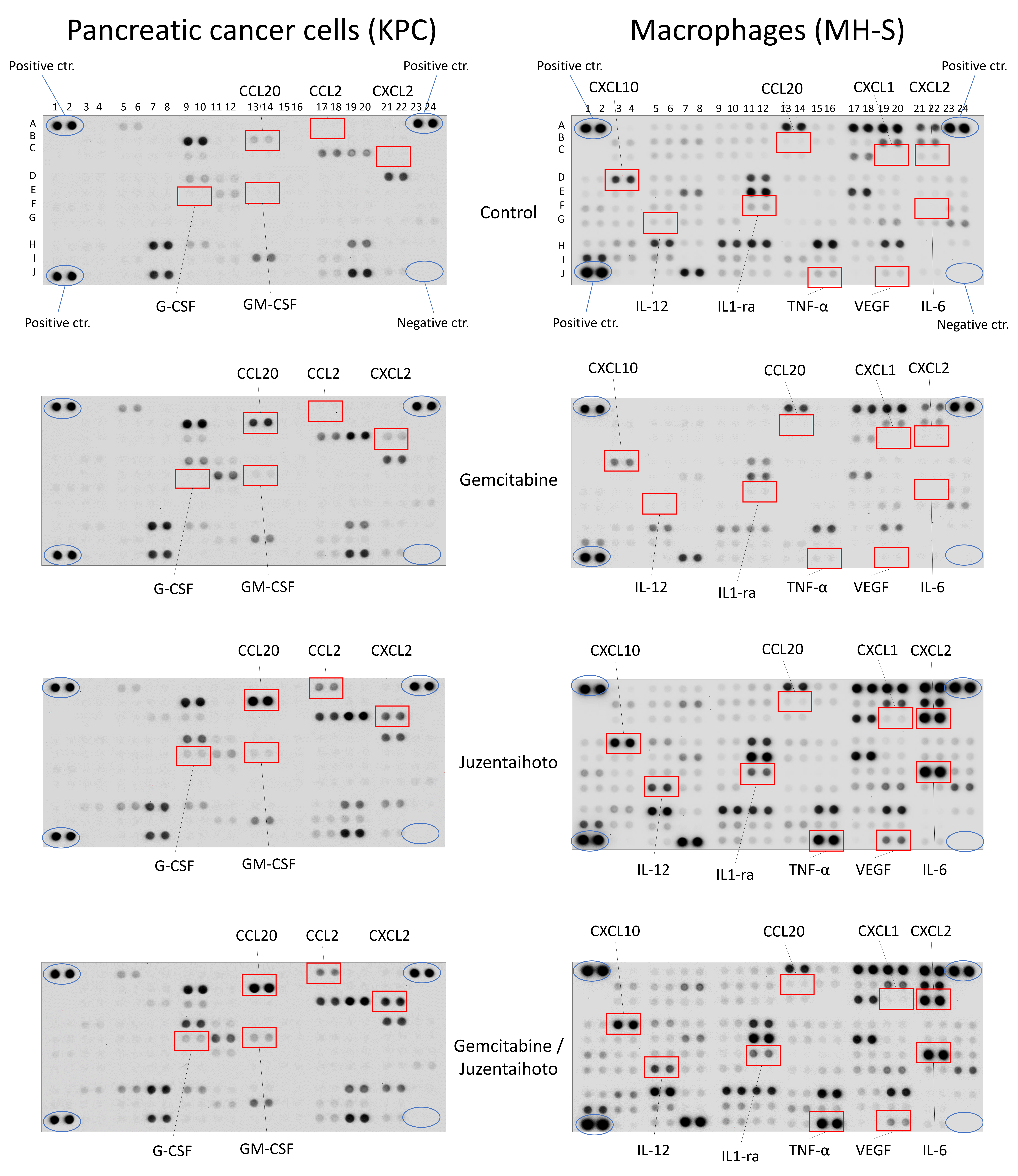

Supplement: Supplementary file 6 [file Image6.tif]
