# Supplementary material for: Prolonged survival by combination treatment with a standardized herbal extract from Japanese Kampo-medicine (Juzentaihoto) and gemcitabine in an orthotopic transplantation pancreatic cancer model
Source: Front Oncol. 2024 Dec 11;14:1454291. doi: 10.3389/fonc.2024.1454291 (PMC11669038; doi:10.3389/fonc.2024.1454291)
Supplement: Supplementary file 8 [file Table1.docx]

| **Transcript** | **Name** | **Sequence** |
| --- | --- | --- |
| CCL2 | For | CAG CCA GAT GCA ATC AAT GCC |
|  | Rev | TGG AAT CCT GAA CCC ACT TCT |
| CCL20 | For | TTT GGC ATG GCT ACT GCT G |
|  | Rev | AGG AGG TTC ACA GCC CTT TT |
| CXCL2 | For | GAA GTC ATA GCC ACT CTC AAG G |
|  | Rev | TTC CGT TGA GGG ACA GCA |
| CXCL10 | For | AAG TGC TGC CGT CAT TTT CT |
|  | Rev | CCT ATG GCC CTC ATT CTC AC |
| GM-CSF | For | ATG CCT GTC ACG TTG AAT GAA G |
|  | Rev | GCG GGT CTG CAC ACA TGT TA |
| IL-6 | For | AGT TGC CTT CGG ACT GA |
|  | Rev | CAG AAT TGC CAT TGC ACA AC |
| IL-10 | For | ACA GTC CAT CAA GGT TAG CAG |
|  | Rev | GCA ACC CAA GTA ACC CTA GAG |
| RPLP0 | For | TGG GCA AGA ACA CCA TGA TG |
|  | Rev | AGT TTC TCC AGA GCT GGG TTG T |
| TGF-ß | For | GGA CTC TCC ACC TGC AAG ACC |
|  | Rev | GGA TGG CTT CGA TGC GC |
| TNF-α | For | TCT CCC CAC ACC AAC TTT TC |
|  | Rev | GGG CTA TCA AGA TCA GAG GTC |

Supplementary Table 1: *Primers used to assess expression of the selected genes by real time PCR. RPLP0 – housekeeping gene (Ribosomal Protein Lateral Stalk Subunit P0).*
